# Identification of microRNAs Actively Involved in Fatty Acid Biosynthesis in Developing *Brassica napus* Seeds Using High-Throughput Sequencing

**DOI:** 10.3389/fpls.2016.01570

**Published:** 2016-10-24

**Authors:** Jia Wang, Hongju Jian, Tengyue Wang, Lijuan Wei, Jiana Li, Chao Li, Liezhao Liu

**Affiliations:** ^1^College of Agronomy and Biotechnology, Southwest UniversityChongqing, China; ^2^Nanchong Academy of Agricultural SciencesNanchong, China; ^3^Guizhou Province Institute of Oil CropsGuiyang, China

**Keywords:** microRNA, *Brassica napus*, seed development, fatty acid biosynthesis, target gene

## Abstract

Seed development has a critical role during the spermatophyte life cycle. In *Brassica napus*, a major oil crop, fatty acids are synthesized and stored in specific tissues during embryogenesis, and understanding the molecular mechanism underlying fatty acid biosynthesis during seed development is an important research goal. In this study, we constructed three small RNA libraries from early seeds at 14, 21, and 28 days after flowering (DAF) and used high-throughput sequencing to examine microRNA (miRNA) expression. A total of 85 known miRNAs from 30 families and 1160 novel miRNAs were identified, of which 24, including 5 known and 19 novel miRNAs, were found to be involved in fatty acid biosynthesis.bna-miR156b, bna-miR156c, bna-miR156g, novel_mir_1706, novel_mir_1407, novel_mir_173, and novel_mir_104 were significantly down-regulated at 21 DAF and 28 DAF, whereas bna-miR159, novel_mir_1081, novel_mir_19 and novel_mir_555 were significantly up-regulated. In addition, we found that some miRNAs regulate functional genes that are directly involved in fatty acid biosynthesis and that other miRNAs regulate the process of fatty acid biosynthesis by acting on a large number of transcription factors. The miRNAs and their corresponding predicted targets were partially validated by quantitative RT-PCR. Our data suggest that diverse and complex miRNAs are involved in the seed development process and that miRNAs play important roles in fatty acid biosynthesis during seed development.

## Introduction

MicroRNAs (miRNAs) are non-coding RNAs of ~22 nucleotides in length that largely negatively regulate the translation of protein-coding gene(s) by binding to perfect complementarity sites in the 3′ untranslated regions (UTRs) of messenger RNAs (mRNAs), thereby targeting transcripts for cleavage or blocking their translation (Tarver et al., 2013). miRNAs are reported to be involved in a broad range of metabolic and physiological processes in plants, such as growth (Jones-Rhoades et al., 2006), development (Rubio-Somoza and Weigel, 2011) and responses to various stresses (Khraiwesh et al., 2012). There gulatory role of miRNAs is exemplified by their critical regulatory behavior at key steps in a variety of pathways, such as root (Wang et al., 2005), shoot (Golz, 2006), leaf (Kidner and Martienssen, 2004), and flower (Teotia and Tang, 2015) development and cell fate (Carraro et al., 2006), and it is likely that their gene regulation function is as critical in maturing seeds as in other tissues(Martin et al., 2006). To date, researchers have been able to identify conserved and novel miRNAs in *Arabidopsis* (659) (Mallory et al., 2005; Reyes and Chua, 2007),rice (1500) (Xue et al., 2009; Zhang et al., 2012; Yi et al., 2013; Peng et al., 2014), maize (158) (Kang et al., 2012; Li et al., 2013), barley (101) (Curaba et al., 2012), wheat (1920) (Meng et al., 2013; Han et al., 2014), soybean(399) (Song et al., 2011) and rapeseed (90) (Xie et al., 2007; Korbes et al., 2012; Zhao et al., 2012) seeds.

Seed production comprises a unique transitional process during the life cycle of higher plants, provides a physical link between parental and progeny sporophytic generations (Meng et al., 2005), and plays an important role in plant survival. Seed development is accompanied by complex physiological and biochemical changes; the most significant events include the accumulation of storage reserves in three major forms: carbohydrates (often starch), lipids in the form of triacylglycerides (TAGs) and storage proteins (Huang, 1992; Mansfield and Briarty, 1992; Goldberg et al., 1994). Understanding the alterations that occur in seeds at different developmental stages and establishing a regulatory network of miRNAs involved in seed development, especially with regard to fatty acid biosynthesis in oil crops, are essential for identifying the mechanism by which miRNAs regulate seed development.

Rapeseed (*Brassica napus* L.) is a major crop with great economic importance due to its seed oil used in human nutrition and protein used in animal feed. During embryogenesis, the vast majority of the reserves of *B. napus* seeds consist of lipids (40–45%) and proteins (17–26%) stored almost exclusively in the cotyledons of the maturing embryo (Appelqvist, 1972). Oil body (lipid-containing structures) biogenesis begins as early as the heart stage during embryogenesis, and lipid accumulation typically starts approximately 3 weeks after flowering and peaks after another 3 weeks (Eastmond and Rawsthorne, 2000; He and Wu, 2009). As an excellent model system for studying seed development, *Brassica* species have become a major focus of plant research regarding the genetic control of seed filling with storage molecules (Purugganan and Fuller, 2009). Indeed, the pathways of fatty acid biosynthesis and seed TAG assembly in oilseed species have been extensively studied (Baud and Lepiniec, 2010; Bates et al., 2013), and it has been described that *de novo* fatty acid biosynthesis occurs in the plastids of developing seeds. However, there are few reports on the miRNAs involved in the regulation of *B. napus* seed oil biosynthesis (Zhao et al., 2012; Deng et al., 2015).

Thus, to systematically identify miRNAs that may be involved in regulating early embryonic development in *B. napus* and seed oil biosynthesis, we constructed small RNA libraries from early developing seeds at 14, 21, and 28 days after flowering (DAF) and profiled small RNA expression using high-throughput sequencing. A total of 85 known miRNAs from 30 families and 1160 novel miRNAs were identified together with their targets. Expression analysis revealed some miRNAs with variable expression levels at different stages of seed development. Our study expands the general understanding of the mechanism by which miRNA regulates gene expression as well as miRNAs that potentially participate in seed development and fatty acid biosynthesis in *B. napus*.

## Materials and methods

### Plant material, small RNA library construction, and RNA sequencing

A *B. napus* double haploid line (F117) with stable oil content over 3 years was used in this study. The plants were grown under natural conditions in the experimental field of the Chongqing Engineering Research Center for Rapeseed, Southwest University in Beibei, Chongqing, China (106.40°E, 29.80°N) from October 2014 to May 2015. Developing seeds from different F117 plants were collected in the middle of a light cycle at 14, 21, and 28 DAF and immediately frozen in liquid nitrogen and stored at −80°C until use. Total RNA was isolated using TRIzol H (Invitrogen, USA) according to the manufacturer's instructions, and the RNA quality was evaluated by electrophoresis on a 1% agarose gel (Han et al., 2014). Total RNA (>10 μg) was sent to Beijing Genome Institute (BGI, Shenzhen, China) for sRNA library construction and Solexa sequencing using standard protocols with the Illumina HiSeq 2000 platform.

### Small-RNA data analysis

Small RNA libraries were constructed and sequenced for the three stages (14, 21, and 28 DAF); all raw sequences were filtered with the SOAPnuke software (http://soap.genomics.org.cn/; Li et al., 2009). Low-quality reads, reads smaller than 18 nt, adaptor sequences, and contamination by adaptor–adaptor ligation were removed according to the software's default settings. The raw sequences were categorized to unique reads and annotated using the Rfam database (http://www.sanger.ac.uk/software/Rfam) and the GenBank non-coding RNA database (http://www.ncbi.nlm.nih.gov/). Small RNAs were then aligned to miRNA precursors of rapeseed in miRBase 21.0 (Kozomara and Griffiths-Jones, 2014), and the expression of known miRNAs was assessed.

To identify novel miRNAs, the software Mireap (http://sourceforge.net/projects/mireap/) developed by BGI was used to predict the unannotated small RNA reads mapping to the *B. napus* genome. A small RNA was regarded as a novel miRNA candidate if it met certain criteria described previously (Wang et al., 2011; Ding et al., 2012). Potential targets for the miRNAs were predicted using the psRobot software with default parameters (Wu et al., 2012). A previously defined scoring system was used to evaluate all predicted target genes, and genes with a score less than 3.0 were considered miRNA targets (Srivastava et al., 2014).

### GO and KEGG pathway analyses

To better understand miRNA target functions and classifications as well as the metabolic regulatory networks associated with *B. napus* miRNAs and their targets, all target genes were mapped to Gene Ontology (GO) terms (http://www.geneontology.org/), and the number of genes for each term was calculated. To identify significantly enriched GO terms, a hypergeometric test was utilized to compare the target gene candidates with the reference gene background to determine the *P*-value (Sha et al., 2014). GO terms with a *P*-value less than the threshold of 0.05 were considered to be significantly enriched. GO annotation results were plotted using WEGO (http://wego.genomics.org.cn/cgi-bin/wego/index.pl). Kyoto Encyclopedia of Genes and Genomes (KEGG, http://www.genome.jp/kegg) was used to analyse metabolic pathway assignments. The test and threshold values for estimating significantly enriched metabolic pathways and signal transduction pathways were the same as those used for the GO analysis (Geng et al., 2015).

### qRT-PCR validation

Quantitative real-time PCR (qRT-PCR) for miRNAs and their targets was performed using a CFX96 Real-time System (BIO-RAD, USA). Total RNA from each sample was extracted as described above. Briefly, 1 μg of RNA from each sample was used to generate single-stranded miRNA cDNA by reverse transcription with miRcute miRNA First-Strand cDNA synthesis Kit (TIANGEN, Beijing, China) and miRNA-specific primers provided with the kit. Next, the expression levels of miRNAs involved in fatty acid biosynthesis were analyzed in three seed developmental stages using qRT-PCR and miRNA-specific primers with a CFX96 Real-time System (BIO-RAD, USA) and SYBR® Premix (TIANGEN, Beijing, China). U6 snRNA was used as the reference gene in qRT-PCR.

Predicted target genes were validated by quantitative RT-PCR using specific primers designed with the software Primer Premier 5.0 (PREMIER Biosoft Int., Palo Alto, CA, USA). qRT-PCR was performed with a CFX96 Real-time System (BIO-RAD, USA) using SYBR® Premix (TIANGEN, Beijing, China). Actin7 was used as an endogenous control. All samples were subjected to three technical replicates.

## Results

### Overview of small RNA library sequencing

**Figure 1:**
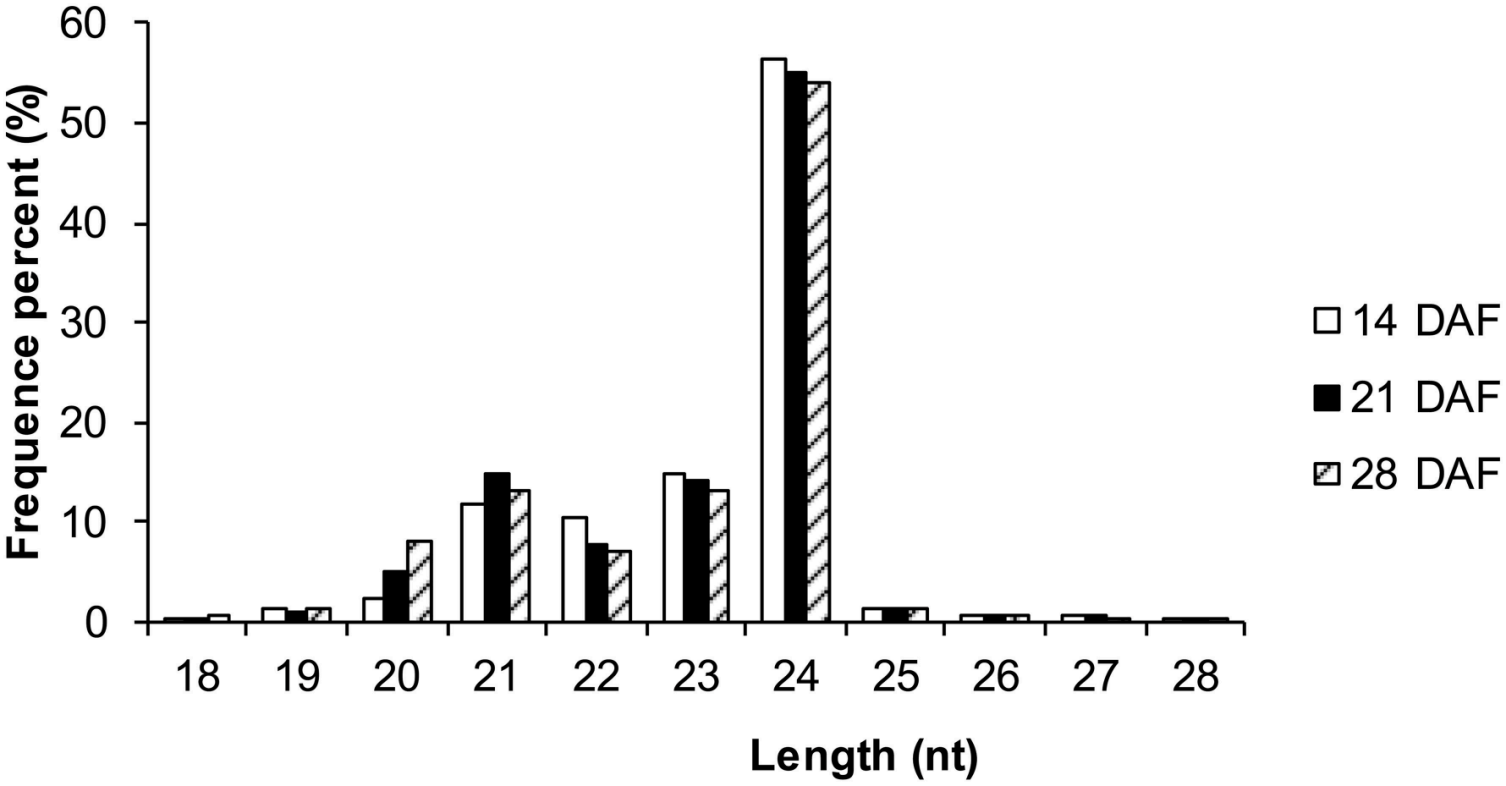
**Length distribution of small RNAs in 14, 21, and 28 DAF ***B. napus*** libraries**.

Deep sequencing of three small RNA libraries from developing *B. napus* seeds produced 12,225,750, 11,419,839, and 11,427,691 raw sequence reads. After the removal of low-quality reads and 3′ adapter, 5′ adapter, corrupted adapter (reads < 10 nt or >30 nt long) and other contaminating sequences, 12,120,056 (99.49%), 11,334,399 (99.63%), and 11,335,373 (99.57%) clean reads were obtained from the 14, 21, and 28 DAF libraries, respectively (Table S1). After the further removal of unannotated small RNAs and non-coding RNAs, such as tRNAs, rRNAs, siRNAs, snRNAs, snoRNAs and other non-coding RNAs, 393,346 (3.25%), 746,228 (6.59%), and 1,083,239 (9.56%) miRNA sequences were identified in the three libraries (14, 21, and 28 DAF, respectively; Table 1). The meaningful feature of the size profile permitted the miRNAs to be distinguished from other small RNAs. The miRNA length distribution (18–28 nt) of the original reads revealed that those 20–24 nt in length were the most abundant (Figure 1).

**Table 1:** **Summary of small RNAs based on sequencing data**.

**Category**	**14 DAF**		**21 DAF**		**28 DAF**	
	**Total**	**Uniq**.	**Total**	**Uniq**.	**Total**	**Uniq**.
intron_antisense	362377 (2.99%)	101034 (3.13%)	335834 (2.97%)	137114 (3.35%)	359567 (3.18%)	155802 (3.48%)
intron_sense	475752 (3.93%)	141298 (4.38%)	527866 (4.66%)	193519 (4.73%)	594692 (5.25%)	221325 (4.94%)
snRNA	2254 (0.02%)	1401 (0.05%)	2912 (0.03%)	1631 (0.04%)	3284 (0.03%)	1946 (0.05%)
exon_sense	1243907 (10.27%)	177806 (5.51%)	778217 (6.87%)	160941 (3.93%)	712209 (6.29%)	166778 (3.72%)
unannotation	8263655 (68.19%)	2622361 (81.23%)	7964692 (70.28%)	3430831 (83.78%)	7535826 (66.49%)	3756616 (83.79%)
rRNA	340666 (2.82%)	37106 (1.15%)	358932 (3.17%)	36401 (0.89%)	463877 (4.1%)	41986 (0.94%)
snoRNA	1269 (0.02%)	785 (0.03%)	1288 (0.02%)	785 (0.02%)	1292 (0.02%)	835 (0.02%)
repeat	3634 (0.03%)	1423 (0.05%)	5145 (0.05%)	2651 (0.07%)	5567 (0.05%)	2951 (0.07%)
exon_antisense	990651 (8.18%)	142003 (4.4%)	570229 (5.04%)	127895 (3.13%)	509445 (4.5%)	131424 (2.94%)
miRNA	393346 (3.25%)	316 (0.01%)	746228 (6.59%)	357 (0.01%)	1083239 (9.56%)	390 (0.01%)
tRNA	42545 (0.36%)	2900 (0.09%)	43056 (0.38%)	3267 (0.08%)	66375 (0.59%)	3729 (0.09%)
Total	12120056 (100%)	3228433 (100%)	11334399 (100%)	4095392 (100%)	11335373 (100%)	4483782 (100%)

### Identification of known miRNA families and novel candidate miRNAs in *B. napus*

**Figure 2:**
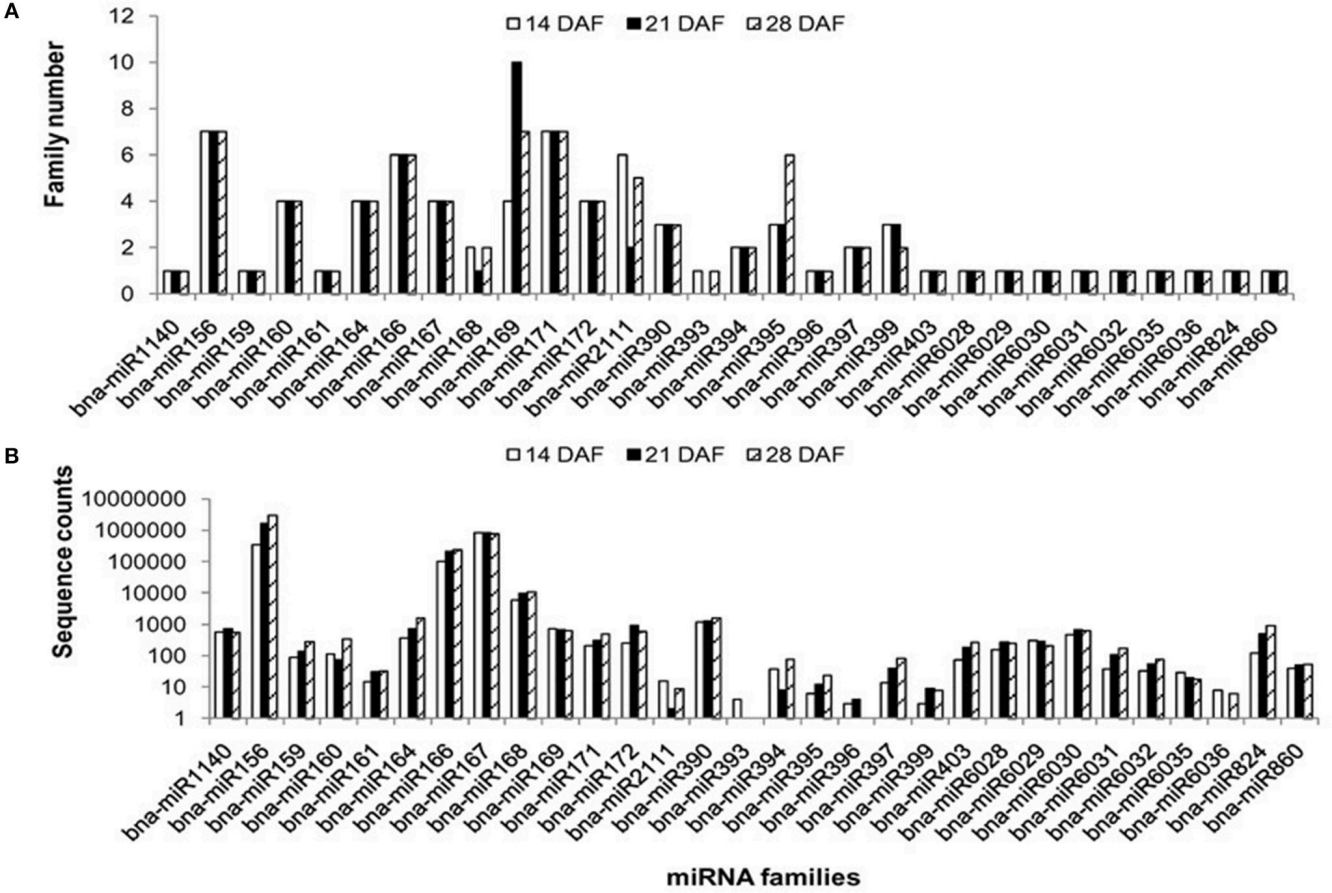
**Sizes and abundance of identified ***B. napus*** miRNA families. (A)** Distribution of known miRNA family size in *B. napus*. **(B)** Counts for each known miRNA family in *B. napus*.

By mapping unique sRNA sequences to miRBase 21.0 with a maximum of two mismatches, a total of 85 unique sequences belonging to 30 known miRNA families were identified in the three libraries. Among the known miRNA families, seven, six, and seven members were found from the miR156, miR166, and miR171 families, respectively. As the most abundant in the 21 DAF library, 10 members of the miR169 family were identified. In addition, only one member was found for 10 miRNA families (Figure 2A; Table S2).

The number of reads differed drastically among the 30 known miRNA families. Extraordinarily high expression levels of a few known miRNA families, such as miR156, miR166, and miR167, were identified in all three libraries. MiR156 was the most abundant, with 343,028 (14 DAF), 1,572,529 (21 DAF), and 2,959,044 (28 DAF) reads accounting for 27.4, 59.7, and 74.1% of all known miRNA reads, respectively (Figure 2B). Several miRNA families, including miR164, miR168, and miR390, exhibited moderate abundance. In contrast, a few known miRNA families, such as miR161, miR393, miR2111, miR395, miR396, miR399, miR6035, and miR6036, showed relatively lower expression levels and were represented by < 50 reads in the three libraries. Among these miRNAs, 69 miRNAs were expressed at all three developmental stages, with only 1, 5, and 3 co-expressed at 14 and 21 DAF, 14 and 28 DAF, 21 and 28 DAF, respectively. For example, bna-miR2111c is stage-specifically expressed only at 14 DAF, bna-miR169i, bna-miR169j and bna-miR169l only at 21 DAF, and bna-miR395a, bna-miR395b and bna-miR395c only expressed at 28 DAF. Sixty-nine miRNAs were expressed at all three developmental stages, some of which demonstrated little variation throughout seed development, suggesting that they perhaps fulfill housekeeping functions.

**Figure 3:**
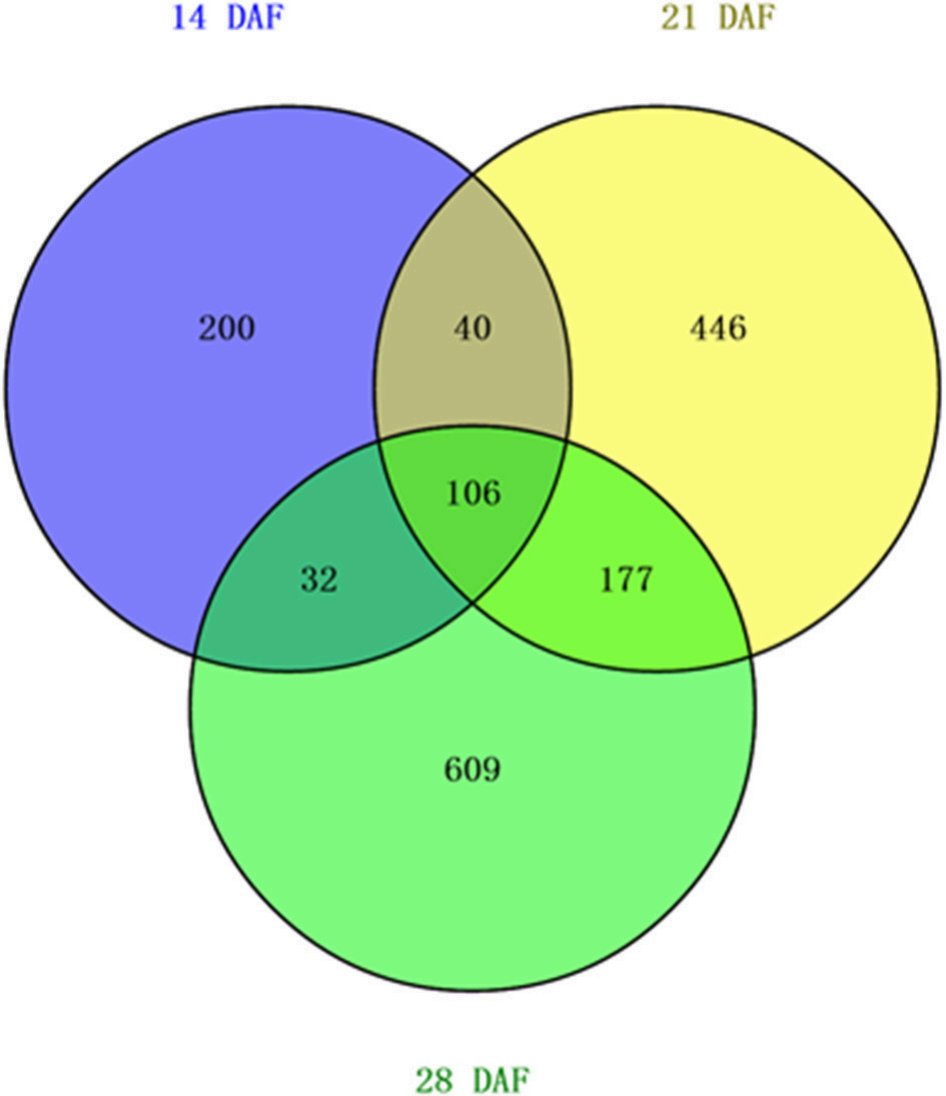
**Distribution of novel miRNAs in 14, 21, and 28 DAF libraries**.

To predict novel miRNAs, Mireap was used with strict criteria (Li et al., 2013) that include the characteristic hairpin structures of miRNA precursors, Dicer cleavage sites, and minimum free energy. In total, 1610 novel miRNAs were predicted from the three libraries; the lengths of the novel miRNAs ranged from 20 to 24 nt, with 24 nt being the most common in all three libraries (Table S3; Figure S1). More than half of the novel predicted miRNAs begin with a 5′ uridine, and these miRNAs accounted for more than 80% of 20 and 21 nt small RNAs. Compared with known miRNA families, the abundance of novel miRNAs was very low, and the majority were present in less than 50 reads. Nonetheless, these miRNAs comprised 83.26% (378/454), 91.01% (769/845), and 92.03% (924/1004) of the 14 DAF, 21 ADF and 28 DAF libraries, respectively. The most abundant novel miRNA was novel_mir_146, which was sequenced in 10,168, 13,227, and 16,128 reads of the 14, 21, and 28 DAF libraries, respectively (Table S2). Unlike known miRNAs, different types of novel miRNAs were expressed in the three independent libraries: 106 miRNAs were expressed at all three developmental stages; 40, 32, and 177 were co-expressed at 14 and 21 DAF, 14 and 28 DAF, 21 and 28 DAF, respectively; 200, 446, and 609 were stage specifically expressed at 14, 21, and 28 DAF, respectively (Figure 3); 106 were expressed at all three developmental stages, some of which showed little variation throughout seed development, suggesting housekeeping functions for these miRNAs.

### Target gene prediction of miRNAs and GO analysis

The identification of miRNA target genes using bioinformatic approaches is essential for understanding the regulatory function of miRNAs (Zhao et al., 2013). In this study, the software psRobot was used to predict miRNA targets of known and novel miRNAs; using a cut-off threshold of 3.0, 2582, and 10,032 putative targets were found, respectively (Table S4). Conversely, no target genes were predicted for the remaining 180 novel miRNAs. Among the known miRNAs identified in our analysis, bna-miR156d, bna-miR156e, and bna-miR156f have 134 putative target genes with different functions, indicating that these three miRNAs are involved in regulating the expression of multiple genes in *B. napus*.

**Figure 4:**
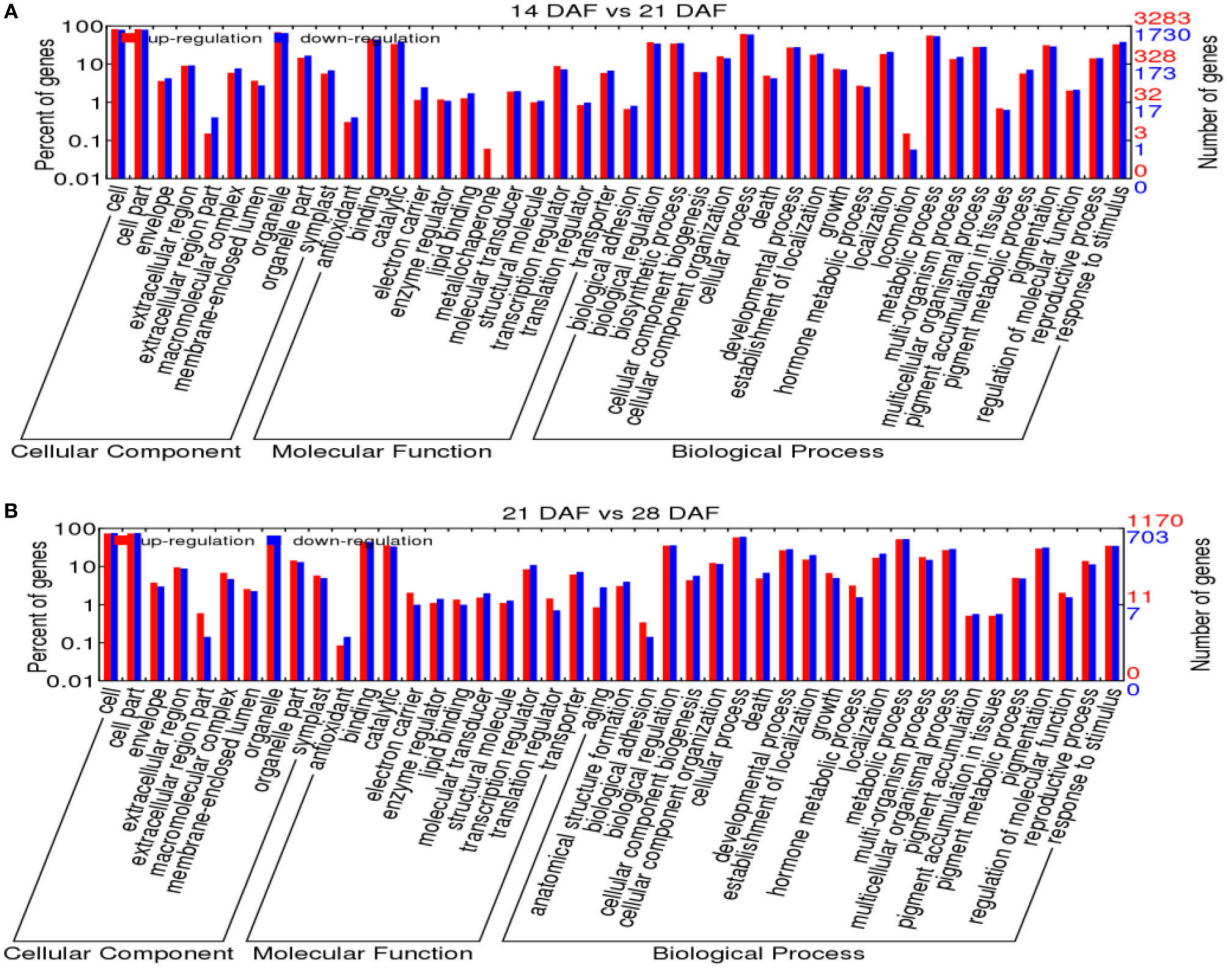
**Gene ontology classification of miRNA targets in seed development**. **(A)** 14 vs. 21 DAF, **(B)** 21 vs. 28 DAF.

Using the criteria of an absolute fold change value ≥1.0 and a *P*-value ≤ 0.05, 702, and 509 miRNAs showed significantly different expression between the 14 and 21 DAF as well as 21 and 28 DAF libraries, respectively. Comparing the 14 and 21 DAF libraries indicated 5013 significantly altered genes, with 3283 up-regulated and 1730 down-regulated, and 1873 genes were detected between the 21 and 28 DAF libraries, with 1170 up-regulated and 703 down-regulated (Tables S2, S4). GO analysis was used to classify the functions of the target genes of the miRNAs differentially expressed during seed development based on the three main categories: biological process, cellular component, and molecular function). For 14 vs. 21 DAF, the target genes are involved in 12 different molecular functions, 22 biological processes, and 10 cellular components, and for 21 vs. 28 DAF, 23 biological processes, 11 molecular functions, and 10 cellular components were identified (Figure 4). Many biological processes were found to be involved, including cellular process (GO: 0009987), biological regulation (GO: 0065007), and metabolic process (GO: 0008152). Figure 4 shows up-regulated target genes specifically enriched during different seed developmental stages, involving amino acid biosynthesis, pigment accumulation, embryonic development and others.

### Expression profiles of miRNAs involved in acetyl-CoA conversion to fatty acids

To illuminate the relationship between miRNA and putative gene function, we constructed miRNA regulatory networks for fatty acid biosynthesis, pigment accumulation, embryonic development, sugar conversion, amino acid metabolism, plant hormones, and signaling pathways during seed development (Figure S2). Following these analysis, KEGG pathway analysis identified significant enrichment of 85 pathways with predominant enrichment of seed development-related pathways (Table S5).

**Figure 5:**
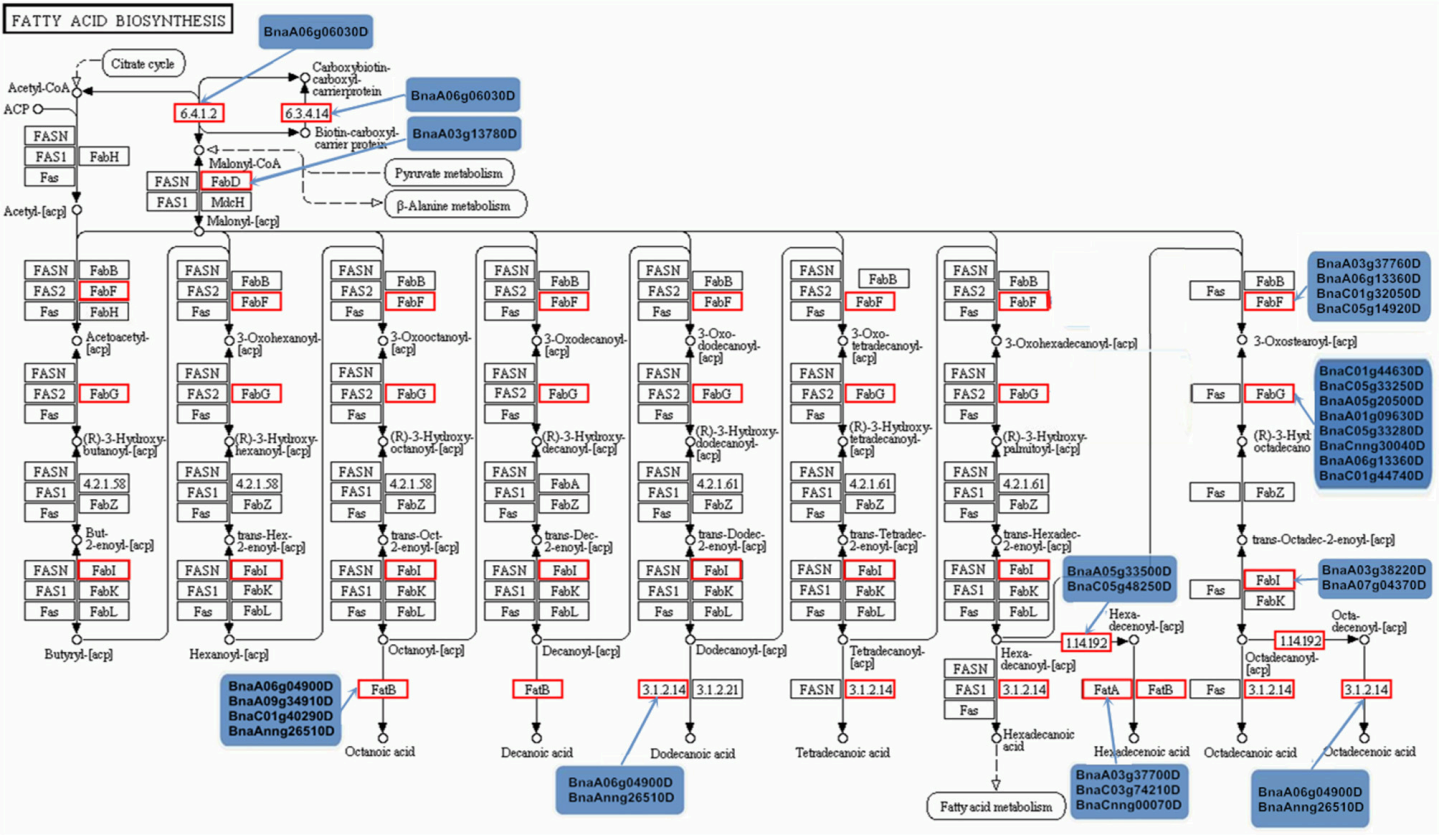
**Targets involved in KEGG pathways of fatty acid biosynthesis**. Red indicates target genes participating in the pathway, and the corresponding targets are shown.

To examine the molecular mechanism of fatty acid biosynthesis during seed development, we investigated target genes related to the fatty acid biosynthesis pathway of differentially expressed miRNAs. As shown in Figure 5, *de novo* synthesis of fatty acids utilizes acetyl-CoA as a substrate and malonyl-ACP as an elongator, and 27 targets, which encode 10 catalytic enzymes, are involved in plastid acetyl-CoA conversion to fatty acids. The formation of malonyl-CoA from acetyl-CoA and bicarbonate by acetyl-CoA carboxylase (ACC) has long been considered a key regulatory step of fatty acid biosynthesis (Turnham and Northcote, 1983; Harwood, 1996), and the miRNA target *BnaA06g06030D* encodes the carboxyltransferase alpha subunit of acetyl-CoA carboxylase (α-CT, EC: 6.4.1.2) and the biotin carboxylase subunit (BC, EC: 6.3.4.14). The malonyl-CoA produced by plastidial ACC constitutes the carbon donor for each cycle of the fatty acid biosynthesis pathway (Hannapel and Ohlrogge, 1988; Bonaventure and Ohlrogge, 2002). Malonyl-thioester undergoes a series of condensation reactions with acetyl-CoA percycle, steps that are catalyzed by 3-ketoacyl-ACP synthase of type III (KASIII, EC: 2.3.1.179), 3-ketoacyl-ACP reductase (KAR, EC: 1.1.1.100), 3-hydroxyacyl-ACP dehydratase (HAD, EC: 4.2.1.59), and enoyl-ACP reductase (ER, EC: 1.3.1.9), to produce a saturated fatty acid with two additional carbons. Among the four steps, 14 targets were found for three steps (EC: 2.3.1.179, EC: 1.1.1.100 and EC: 1.3.1.9); four targets (*BnaA03g37760D, BnaA06g13360D, BnaC01g32050D*, and *BnaC05g14920D*) encode KASIII, eight targets encode KAR, and two targets (*BnaA03g38220D* and *BnaA07g04370D*) encode enoyl-ACP reductase. However, none of the targets of differentially expressed miRNAs encode 3-hydroxyacyl-ACP dehydratase. After 7 cycles, the saturated 16-carbon acyl-ACP can either be hydrolysed by FATB acyl-ACP thioesterase (EC: 3.1.2.14; *BnaA06g04900D* and *BnaAnng26510D*) or further elongated by KASII to generate 18:0-ACP, which is then desaturated to 18:1-ACP by stearoyl-acyl-carrier-protein desaturase (EC: 1.14.19.2; *BnaA05g33500D* and *BnaC05g48250D*) and hydrolysed by FATA thioesterase (EC: 3.1.2.14; *BnaA06g04900D* and *BnaAnng26510D*) (Bates et al., 2013).

**Figure 6:**
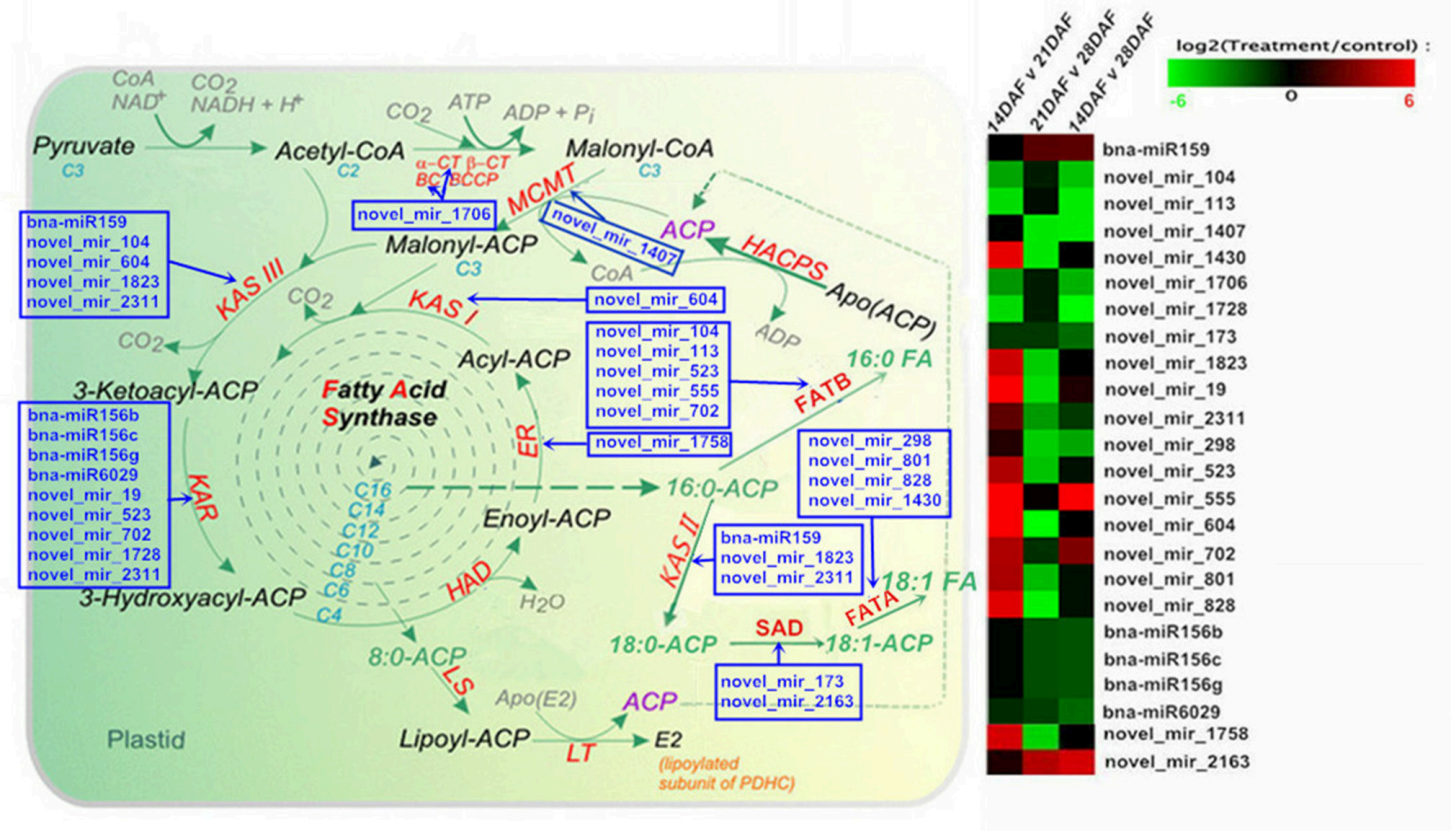
**Analysis of pathways related to acetyl-CoA conversion to fatty acids; the map displays selected steps from KEGG pathways of fatty acid biosynthesis**. Colors indicate miRNAs that differed significantly in expression, with red representing up-regulation and green representing down-regulation (the original drawings of fatty acid biosynthesis are from http://aralip.plantbiology.msu.edu/pathways/fatty_acid_synthesis).

We further explored differentially expressed miRNAs associated with fatty acid biosynthesis, fatty acid desaturation, and fatty acid elongation pathways during seed development. As shown in Figure 6, 24 miRNAs (Table 2), which regulate 10 catalytic enzyme-encoding genes, are involved in the plastid fatty acid biosynthesis pathway. Among these catalytic enzymes, expression of KASII and KASIII is regulated by known the miRNA bna-miR159. The gene encoding KAR is regulated by known the miRNAs bna-miR156b, bna-miR156c, bna-miR156g, and bna-miR6029. Novel miRNAs are found at all 10 steps of the fatty acid biosynthesis pathway, among which KAR and fatty acyl-ACP thioesterases B (FATB) with five miRNAs have the greatest number of regulating miRNAs. In contrast, acetyl Co-enzyme, carboxylase biotin carboxylase subunit (BC), the carboxyltransferase alpha subunit of acetyl-CoA carboxylase (α-CT), malonyl CoA-acyl carrier protein transacylase (MCMT), KASI, and enoyl-ACP reductase (ER) are regulated by a single miRNAs. According to our sequencing results, the expression levels of bna-miR159, novel_mir_19, novel_mir_555, novel_mir_702, and novel_mir_2163 increased significantly, possibly indicating positive regulatory roles for these miRNAs. However, the expression levels of other miRNAs were negatively correlated with the content and composition of fatty acids during the middle and late seed developmental stages, indicating negative regulatory roles. In addition, 16 miRNAs regulating 5 catalytic enzyme-encoding genes are involved in the fatty acid desaturation and fatty acid elongation pathways (Figure S3); steadily up-regulated at 21 DAF and 28 DAF, bna-miR395d, bna-miR395e, and bna-miR395f, which regulate 3-ketoacyl-CoA synthase (KCS), may be involved in fatty acid elongation.

**Table 2:** **Novel miRNAs regulating 11 catalytic enzymes are involved in fatty acid biosynthesis**.

**miRNA**	**Mature sequence (5′-3′)**	**Size**	**LP**	**MFE**	**Target gene**	**Target annotation**
novel_mir_104	GAGGAAGAAGAAGAAGAAGAAGCG	24	90	−21.3	*BnaA03g37760D*	Beta-ketoacyl synthase
novel_mir_555	AAGGAAGAAGAAGAAGATGTAATT	24	76	−23.4	*BnaC01g40290D*	fatty acyl-ACP thioesterases B (FATB)
novel_mir_173	ATCTTGTCGGAGTTTATGATC	21	85	−27.2	*BnaA05g33500D*	Plant stearoyl-acyl-carrier-protein desaturase family protein
novel_mir_19	AGGACCTGATTGCAATGATAACGG	24	90	−18.2	*BnaA01g21430D*	NAD(P)-binding Rossmann-fold superfamily protein
novel_mir_2311	AGGAGAGATTGGATATCCGAACGG	24	82	−23.7	*BnaA06g13360D*	fatty acid biosynthesis 1 (FAB1)
novel_mir_523	AGAGGGGTTGGGGTCGGTGCG	21	90	−68.4	*BnaA09g34910D*	NAD(P)-binding Rossmann-fold superfamily protein
novel_mir_702	ATCGAAAACTTTGACTGATGTGCC	24	84	−20.5	*BnaC01g40290D*	NAD(P)-binding Rossmann-fold superfamily protein
novel_mir_828	GGATCGAATCCAGATCTCGGATA	23	96	−42.9	*BnaC03g74210D*	fatA acyl-ACP thioesterase (FaTA)
novel_mir_2163	AAGGGGATGATTGGTAAGTGCTGT	24	94	−38	*BnaA05g35320D*	SUPER SENSITIVE TO ABA AND DROUGHT2 (SAD2)
novel_mir_801	GATCGAATCCAGATCTCGGATAAA	24	83	−25.8	*BnaA03g37700D*	fatA acyl-ACP thioesterase (FaTA)
novel_mir_298	TTAAGAGATATAAGAACCGTCTAT	24	87	−28.2	*BnaCnng00070D*	fatA acyl-ACP thioesterase (FaTA)
novel_mir_604	AGAGATGGCAATCATGGACTTGGA	24	97	−27.1	*BnaC01g32050D*	3-ketoacyl-acyl carrier protein synthase I (KAS I)
novel_mir_113	TTCCGTCAGAATTTCCTCGGTA	22	90	−28.7	*BnaA06g04900D*	fatty acyl-ACP thioesterases B (FATB)
novel_mir_1407	AGATTAGTCGGTTGGGCTTCGGCC	24	87	−20.6	*BnaA03g13780D*	Malonyl CoA-acyl carrier protein transacylase
novel_mir_1430	GGATCGAATCCAGATCTCGGATAA	24	98	−42.9	*BnaA03g37700D*	fatA acyl-ACP thioesterase (FaTA)
novel_mir_1706	GAGAGTTCGACGGCTAGGGT	20	82	−26.4	*BnaA06g06030D*	acetyl Co-enzyme a carboxylase biotin carboxylase subunit (CAC2)
novel_mir_1728	TCCGAACCGATCTGAACCCGACA	23	97	−21.4	*BnaA08g19070D*	NAD(P)-binding Rossmann-fold superfamily protein
novel_mir_1823	ATTTGGATTGGAGAAAGAGGGTAT	24	100	−19.7	*BnaC05g14920D*	fatty acid biosynthesis 1 (FAB1)
novel_mir_1758	GGAGGAGACGGAGGAGGAGGAGGA	24	101	−31.1	*BnaAnng14470D*	Thioesterase superfamily protein

*LP, length of precursor; MFE (kcal/mol), minimal folding free energy*.

**Figure 7:**
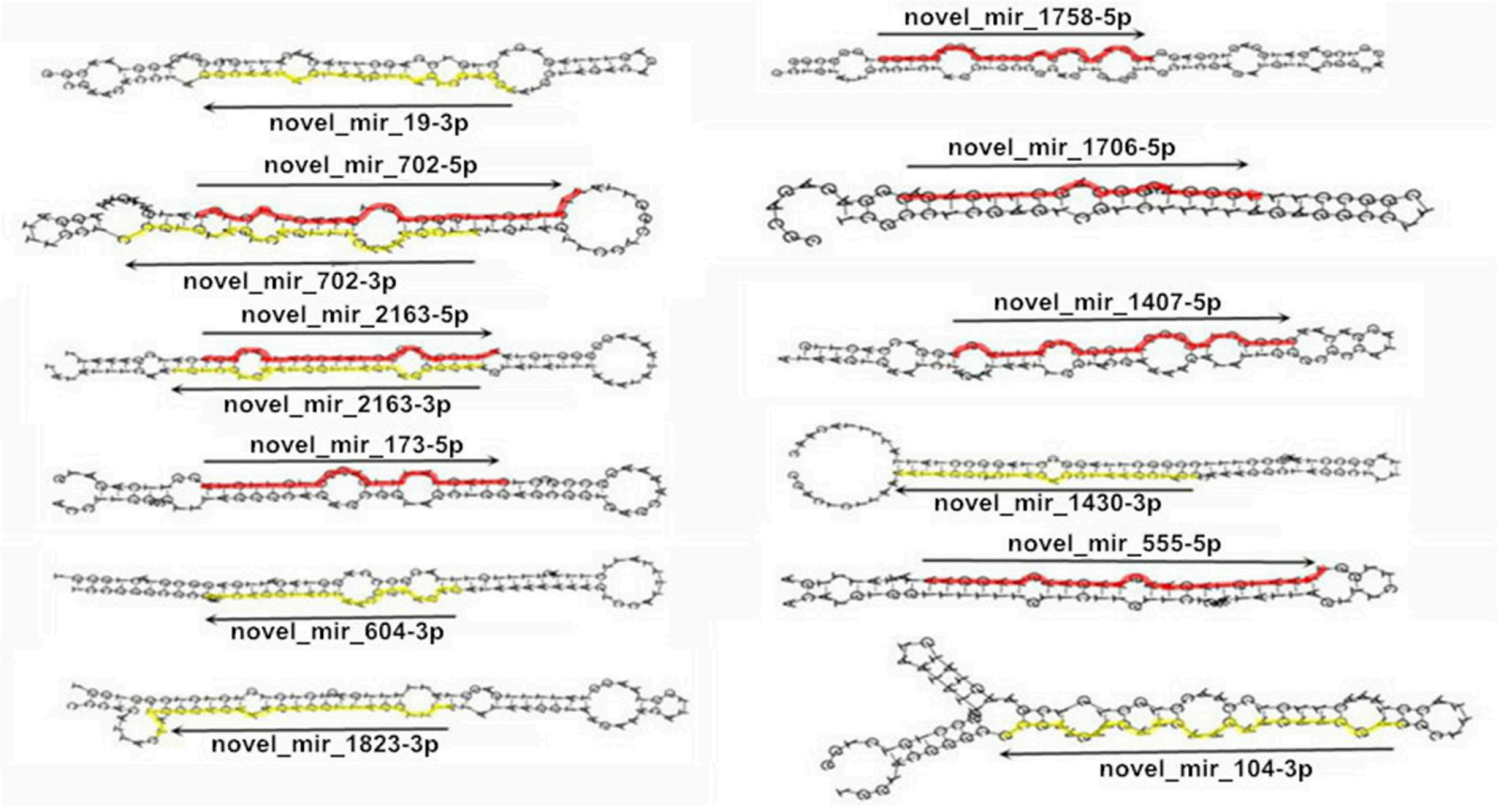
**Mature and precursor sequences and the predicted stem-loop structures of newly identified miRNAs involved in fatty acid biosynthesis in ***B. napus*****. The mature miRNAs are in red (5p) and yellow (3p).

Compared with the size of other miRNA precursors (typically 40–200 nt) reported in a previous study (Xie et al., 2007), the novel miRNA precursors in our work are more diverse in structure but smaller in size (Table 2; Figure 7). The length of miRNA precursors involved in fatty acid biosynthesis varied from 76 to 101 nt, with an average of 89.9 ± 7.1, and approximately 89.5% of these precursors are 80–100 nt in length. The differences in size of the identified miRNAs within different families suggest that they may carry out unique functions in regulating miRNA biogenesis or gene expression (Zhang et al., 2006). The more diverse in structure include novel_mir_702 and novel_mir_2163, which are simultaneously located at the 5′ and 3′ ends of miRNA precursors; in contrast, novel_mir_1407, novel_mir_1706, novel_mir_173, novel_mir_1758, and novel_mir_555 are located at the 5′ end and the others at the 3′ end of miRNA precursors (Figure 7).

### miRNA-regulated genes indirectly involved in fatty acid metabolism

**Figure 8:**
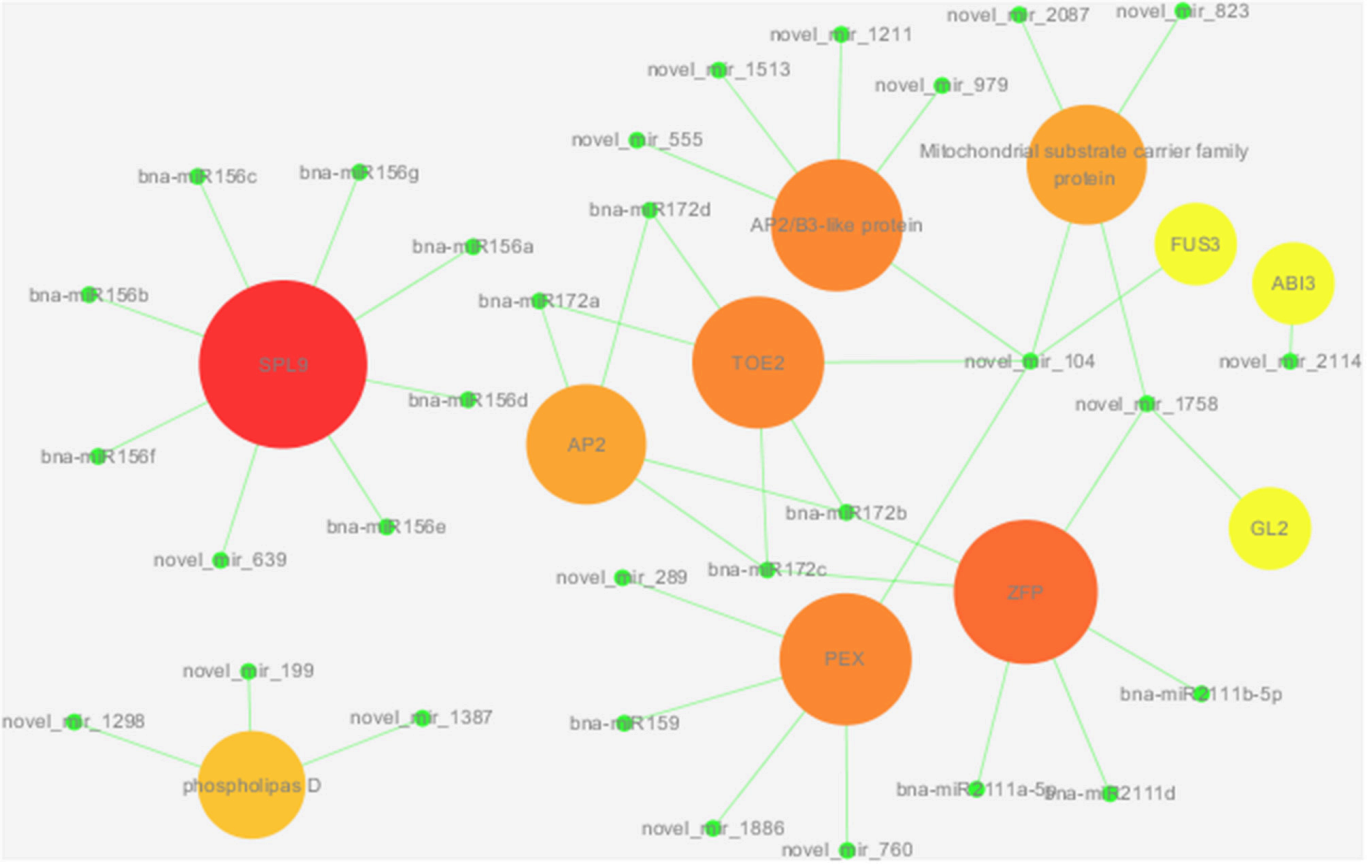
**A miRNA–gene subnetwork built according to interactions between miRNAs and genes**. Green circle nodes denote miRNAs, and the circle nodes of other colors denote genes.

In addition to the miRNAs described above, which may regulate functional genes directly involved in KEGG pathway fatty acid biosynthesis, certain other miRNAs are indirectly involved in fatty acid metabolism by regulating a large number of transcription factors. To understand the regulatory mechanism of miRNAs indirectly involved in fatty acid metabolism, we constructed a miRNA-mediated gene regulatory network for 31 miRNAs and their 11 targets (Figure 8). We analyzed the connection distribution of the network and found that SPL9 and ZFP have the highest number of connections (8 and 6, respectively); ZFP is co-regulated by the bna-miR2111 family, miR172 family and novel_mir_1758, and SPL9 is mainly regulated by the miR156 family. Interestingly, three members of the miR156 family (bna-miR156b, bna-miR156c, and bna-miR156g) directly participate in the regulation of fatty acid biosynthesis; this was also found for transcription factor PEX, which is regulated by bna-miR159 and four novel miRNAs. In addition, the miR172 family regulates targets AP2 and TOE2, and novel_mir_1758 participates in the regulation of GL2 and a mitochondrial substrate carrier family protein. As a key connection, novel_mir_104 regulates five targets. Co-regulated targets of different novel miRNAs can also be observed. Of 11 targets, 10 are regulated by novel miRNAs; more novel miRNAs are involved in the miRNA-mediated gene regulatory network of fatty acid metabolism.

### qRT-PCR validation of miRNAs and corresponding target genes

**Figure 9:**
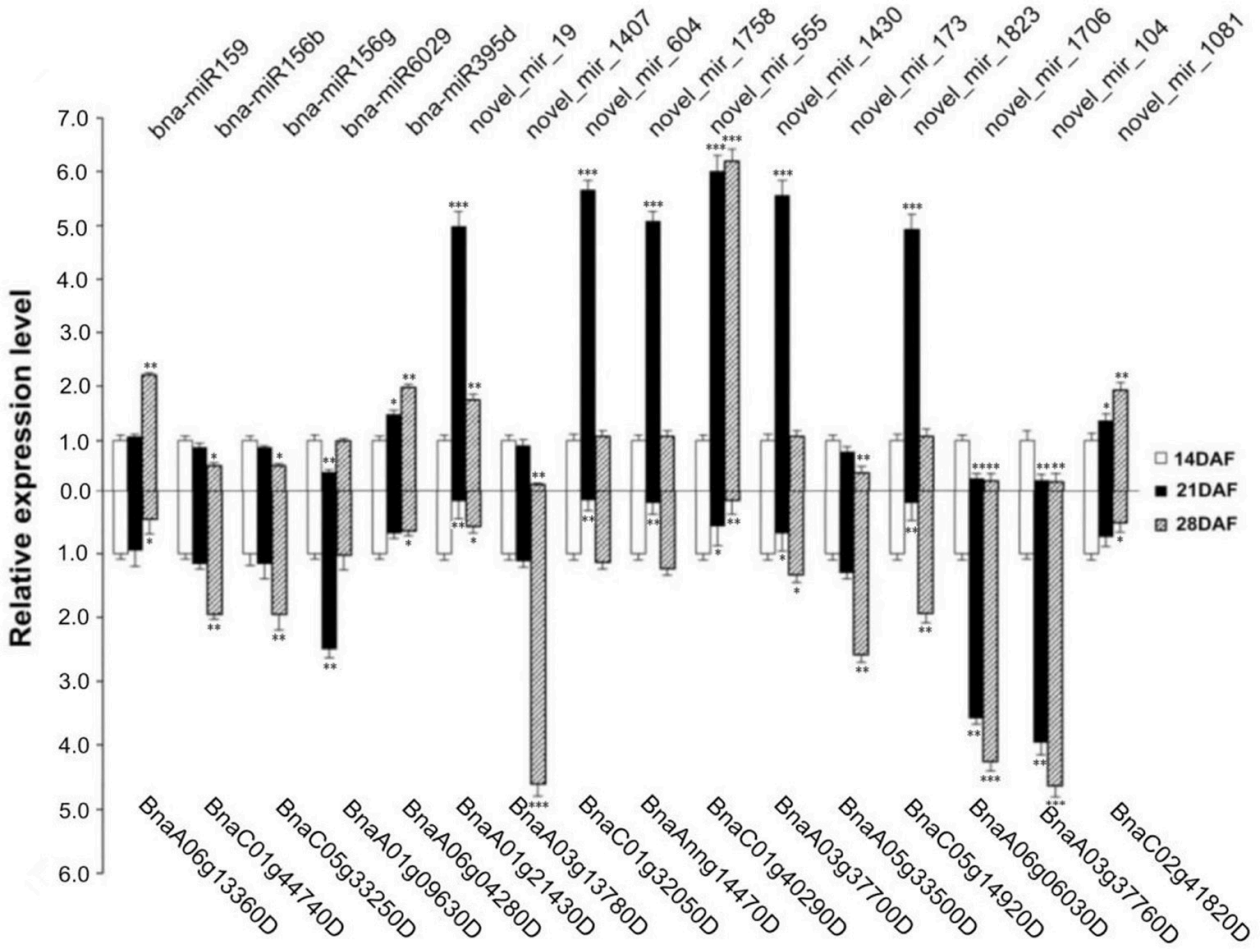
**qRT-PCR validation of selected miRNAs and target genes involved in fatty acid biosynthesis in ***B. napus*****. Upper part and lower part represent miRNAs and target genes, respectively. Small RNAs were extracted from *B. napus* developing seeds from the middle of the main inflorescence (14, 21, and 28 DAF). The normalized levels at 14 DAF were arbitrarily set to 1. ^*^, ^**^ and ^***^ denote significant at the 0.05, 0.01 and 0.001 probability levels, respectively.

To confirm the sequencing results and examine the dynamic expression patterns of the miRNAs involved in fatty acid biosynthesis at different stages of seed development (14, 21, and 28 DAF) in *B. napus*, the expression patterns of five known and 11 novel miRNAs and their corresponding predicted targets were validated by qRT-PCR (Tables S6, S7; Figure S4). As expected, the qRT-PCR data of miRNAs showed a high degree of agreement with the expression profiles obtained by sRNA sequencing of the 14, 21, and 28 DAF libraries, and the qRT-PCR data of the corresponding target gene was just the opposite (Table S2; Figure 9). Regarding known miRNAs, transcripts of bna-miR159 and bna-miR395d were up-regulated, steadily increasing at 21 and 28 DAF; this was particularly obvious for bna-miR159, which exhibited expression changes greater than 2.2-fold between 14 DAF and 28 DAF (*p* < 0.01). By contrast, the gene encoding KASII, FAB1 (*BnaA06g13360D*), a target of bna-miR159, was down-regulated, gradually declined at 21 and 28 DAF, and had greater than 2-fold expression changes between 14 and 28 DAF. Moreover, bna-miR6029 first sharply declined at 21 DAF and then increased at 28 DAF. Novel_mir_1407, novel_mir_173, novel_mir_1706 and novel_mir_104 were down-regulated, remaining at an extremely low expression level at the three stages. The other miRNAs were sharply increased at 21 DAF and then sharply declined at 28 DAF, except that novel_mir_555 was slightly increased at 28 DAF (*p* < 0.001). On the contrary, *BnaA01g09630D* first sharply increased at 21 DAF (*p* < 0.01) and then declined at 28 DAF. *BnaA03g13780D, BnaA05g33500D, BnaA06g06030D* and *BnaA03g37760D* were up-regulated, remaining at an extremely high expression level at the three stages; among these, *BnaA03g37760D* was up-regulated at 28 DAF to 5 times the level at 14 DAF, *BnaA06g06030D* and *BnaA03g13780D* were in the same situation.

## Discussion

MiRNAs act as a key post-transcriptional factors regulating the expression of many genes related to seed development. An increasing number of miRNAs related to seed development are being identified by high-throughput sequencing, and these miRNAs and their target genes comprise a larger network regulating seed development (Li and Zhang, 2016). Using high-throughput technology, we found evidence for the involvement of 85 known miRNAs from 30 miRNA families as well as 1610 novel miRNA at stages of different seed development in *B. napus*. With 10 members, the miR169 family was the largest one found, followed by miR156 (7 members) and miR166 (6 members); 10 families were represented by a single member. The results were similar previous studies, though with some notable differences. Korbes et al. found 172 miRNAs from 45 miRNA families by deep sequencing in a library of developing *B. napus* seeds, with miR156/157 being the largest family (24 members), followed by the miR165/166 (21 members) and miR169 (15 members) families; of the remaining miRNA families identified, between 2 and 6 members were found for 19, and 17 were represented by a single member (Korbes et al., 2012). In addition, we found the shared miRNAs to be highly expressed, whereas the library-specific small RNAs had low levels of expression. Compared with the known miRNA families, the abundance of novel miRNAs was very low, and the majority of these miRNAs were present in less than 50 reads, which has also been observed in maize (Li et al., 2013). The low expression levels of these specific unique small RNAs suggest that they function in specific and unique regulation pathways.

The seed development process of rapeseed directly determines the oil content and quality of the end product. To improve oil content, researchers must have overall understanding of the molecular mechanisms that modulate those steps of fatty acid biosynthesis in seed development. Using transcriptional profiling of developing canola embryos by RNA-sequencing (RNA-Seq), Deng et al. (2015) found that *BnDof 5.6* is required for embryo development and fatty acid biosynthesis (Deng et al., 2015). In addition, the expression profile of the gene encoding sucrose synthase, which increased in the early-stage embryo but gradually decreased in middle- and late-stage embryos, was consistent with starch accumulation in the rapeseed embryo, and the gene encoding plastidial pyruvate kinase demonstrated high expression in both early- and middle-stage embryos. These authors speculated that the genes encoding sucrose synthase and pyruvate kinase may have important roles in starch accumulation and fatty acid synthesis in rapeseed embryos. Zhao et al. (2012) systematically analyzed small RNA expression profiles in *B. napus* seeds at early embryonic stages in high-oil-content and low-oil-content cultivars, both cultured in two environments, identifying 50 conserved and 9 new miRNAs. MiR156 was found in higher abundance in the H cultivar than in the L cultivar, and miR6029 was more highly expressed in the L cultivar than in the H cultivar in both environments. Four miRNA families, namely, miR169, miR390, miR394, and miR6028, had consistently higher expression in the L cultivar, whereas miR408 and miR2111 were more abundant (1.5-fold change cut-off) in the H cultivar in both environments. These results differ slightly from those of our study. For example, bna-miR156, which regulates early embryo development through its target gene *SPL* and thus affects the seed oil content of *B. napus* (Palatnik et al., 2003; Nodine and Bartel, 2010; Wang S. et al., 2012), was the most abundant miRNA family and was gradually down-regulated at 21 and 28 DAF. This result further confirmed that bna-miR156 is involved in regulating seed development and fatty acid biosynthesis in *B. napus*. Bna-miR159, induced by ABA, is involved in controlling the transcript levels of two MYB factors during seed germination and seed development (Reyes and Chua, 2007; Peng et al., 2014); however, little is known about miR159-mediated regulation fatty acid biosynthesis in developing *B. napus* seeds. We found that bna-miR159 is highly but differentially expressed in our three seed libraries. The gene encoding KASII, *FAB1* (*BnaA06g13360D*), a target of bna-miR159, was down-regulated, gradually declined at 21 and 28 DAF, and had greater than 2-fold expression changes between 14 and 28 DAF. Additionally, bna-miR6029, which regulates the gene encoding KAR, was sharply reduced at 21 DAF and then increased at 28 DAF. These results indicate that miR159 and bna-miR6029 have important roles in fatty acid biosynthesis during seed development.

The accumulation of dry matter and lipids for storage reserves is of vital importance to developing *B. napus* seeds, a large number of target genes participate in these processes. Troncoso-Ponce et al. used pyrosequencing to analyse more than seven million ESTs from four stages of developing seeds of four different oilseeds and concluded that high synthetic lipid activity correlates with these developmental stages as does a decline in the expression of genes coding for oil biosynthetic and glycolytic enzymes but not of genes involved in the later steps of oil accumulation (Troncoso-Ponce et al., 2011). In contrast, our results showed these developmental stages are associated with high expression of genes coding for fatty acid biosynthesis enzymes, especially KAR and KASIII because our last sample was collected 28 DAF. GO annotation analyses suggested that miRNAs more abundantly present in developing seeds are most likely involved in up-regulating genes, namely genes related to catalytic enzymes (ACCase, FAS) or essential transcription factors in the regulation of seed development (MYB, SPL, NAC, ABI3). It should be noted that there are some deficiencies in our study, as we focused mainly on activities between 14 and 28 DAF, the key period for lipid accumulation because lipid accumulation usually starts approximately 3 weeks after flowering and peaks after another 3 weeks (Eastmond and Rawsthorne, 2000; He and Wu, 2009; Jolivet et al., 2011).

*B. napus* is one of the most important oil crops in the world, and work on the regulation of fatty acid biosynthesis and the selection of the key regulatory factors for controlling the production of specific fatty acids is among the most important basic research in *B. napus*. Similar to other pathways (Bi et al., 2015), fatty acid synthesis and metabolism are regulated by miRNAs through the activation/inhibition of different important functional genes, and thus miRNAs participate in seed development. Studies have shown that both *FUS3* and *ABI3* are upstream of *WRINKLED1*, which belongs to the B3 family of transcription factors and encodes an AP2/EREB domain transcription factor. *WRINKLED1* is reported to regulate seed oil content because the seed oil content in the *wri1* mutant is significantly decreased, which may be due to decreases in key glycolytic enzyme activity, leading to the accumulation of sucrose and glucose that can't participate in triacylglycerol (Focks and Benning, 1998; Cernac and Benning, 2004; To et al., 2006; Li et al., 2015). Therefore, novel_mir_104 and novel_mir_2114 may be involved in the regulation of lipid metabolism by regulating *FUS3* and *ABI3*, respectively. It has been reported that miR172 targets and promotes the expression of *SPL* genes, which are negative regulators of miR156. MiR156 and miR172 exhibit contrasting development-specific expression patterns: the abundance of miR156 increases during seed development, whereas that of miR172 declines. Data suggest that miR156 is likely to be involved in the metabolism of fatty acids by regulating *SPL9* (Wang et al., 2009; Wu et al., 2009). Interestingly, bna-miR156b, bna-miR156c, and bna-miR156g not only regulate *SPL9*, which is indirectly involved in oil accumulation, but also KASIII, which is directly involved in fatty acid biosynthesis. Zinc finger protein (ZFP), another predicted target gene of bna-miR172b and bna-miR172c, is reported to be associated with fatty acid synthesis and metabolism (Li and Cronan, 1992). In addition, mitochondria substrate carrier family protein, regulated by novel_mir_104, novel_mir_2087, and novel_mir_823, is also reported to be involved in fatty acid biosynthesis (Himms-Hagen and Harper, 2001). Moreover, *GLABRAZ* (*GL2*), a homeobox gene, can regulate oil accumulation in seeds, and the *gl2* deletion mutant displays a high-oil phenotype (Shen et al., 2006); a s*GL2* is regulated by novel_mir_1758, this miRNA is may regulate the lipid accumulation indirectly. According to a previous report, phospholipase D promotes the generation of free fatty acids in plant cells (Wang G. et al., 2012), suggesting that novel_mir_1298, novel_mir_1387, and novel_mir_199 may inhibit the formation of free fatty acids in fruit by regulating phospholipase D, which is important to the formation and accumulation of oil in seeds. PEX is a peroxisome composition factor homolog encoded by the *Shrunken Seed1* (*SSE1*) gene; the oil content of *sse1* mutant seeds is decreased but the starch content significantly increased (Lin et al., 2004), and PEX is regulated by five miRNAs, including bna-miR159, which directly regulates fatty acid biosynthesis functional genes. Furthermore, certain miRNAs showing significantly increased expression may serve a positive regulatory function, and the expression of other miRNAs was negatively correlated with the content and composition of fatty acids during middle and late seed developmental stages, possibly with a negative regulatory role. These results reveal that some miRNAs may regulate functional genes directly involved in fatty acid biosynthesis, whereas other miRNAs regulate the fatty acid biosynthetic process by acting on a large number of transcription factors. Different miRNAs function at different steps via different regulation routes to co-regulate fatty acid biosynthesis. Our study expands our understanding of the molecular mechanism of seed development and fatty acid biosynthesis in *B. napus*.

## Conclusions

Using high-throughput sequencing, we identified 1610 novel miRNAs and 85 known miRNAs belonging to 30 families in the developing seeds of *B. napus*, among which 5 and 19 novel miRNAs were found to be involved in fatty acid biosynthesis. Bna-miR156b, bna-miR156c, bna-miR156g, novel_mir_1706, novel_mir_1407, novel_mir_173, and novel_mir_104 were significantly down-regulated and bna-miR159, novel_mir_1081, novel_mir_19, and novel_mir_555 significantly up-regulated. In addition, we found that some miRNAs regulate functional genes directly involved in fatty acid biosynthesis and that other miRNAs indirectly regulate the fatty acid biosynthesis process through transcription factors. These miRNAs and target genes involved fatty acid synthesis were validated by qRT-PCR.

## Author contributions

CL and LL designed study; JW, HJ, TW, and LW conducted study and analyzed data; CL, LL, and JL provided resources; JW wrote manuscript. All authors read and approved the final manuscript.

## Funding

This work was supported by grants from the National Natural Science Foundation of China (31260337, 31371655).

### Conflict of interest statement

The authors declare that the research was conducted in the absence of any commercial or financial relationships that could be construed as a potential conflict of interest.

## Supplementary material

Figure S1 **Size distribution of novel miRNAs among rapeseed 14, 21, and 28 DAF libraries**.

Figure S2 **miRNA-mediated gene regulatory networks in developing seeds**.

Figure S3 **Analysis of pathways related to fatty acid desaturation and fatty acid elongation**. The original drawings of fatty acid biosynthesis are from http://aralip.plantbiology.msu.edu/pathways/fatty_acid_synthesis.

Figure S4 **A biological replicates for qRT-PCR validation of selected miRNAs**. F117, a *B. napus* double haploid line with stable oil content; F100, a *B. napus* double haploid line with unstable oil content; ZS11, zhongshuang11.

Table S1 **Raw data analysis for the three libraries**.

Table S2 **The abundance of known miRNA families at 14, 21, and 28 DAF**.

Table S3 **Information about novel miRNAs in rapeseed 14, 21, and 28 DAF libraries**.

Table S4 **Predicted targets of known miRNAs and novel miRNAs in the 14, 21, and 28 DAF libraries**.

Table S5 **Significantly enriched KEGG pathways of target genes for differentially expressed miRNAs**.

Table S6 **Primers used for mature miRNA qRT-PCR**.

Table S7 **Primers used for predicted miRNA target qRT-PCR**.
