# Supplementary figures and images for: Identification of microRNAs Actively Involved in Fatty Acid Biosynthesis in Developing Brassica napus Seeds Using High-Throughput Sequencing
Source: Front Plant Sci. 2016 Oct 24;7:1570. doi: 10.3389/fpls.2016.01570 (PMC5075540; doi:10.3389/fpls.2016.01570)

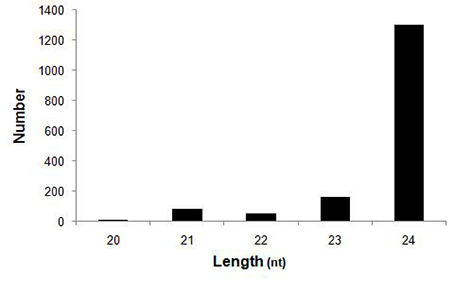

Supplement: Figure S1 — Size distribution of novel miRNAs among rapeseed 14, 21, and 28 DAF libraries. [file Image1.TIF]

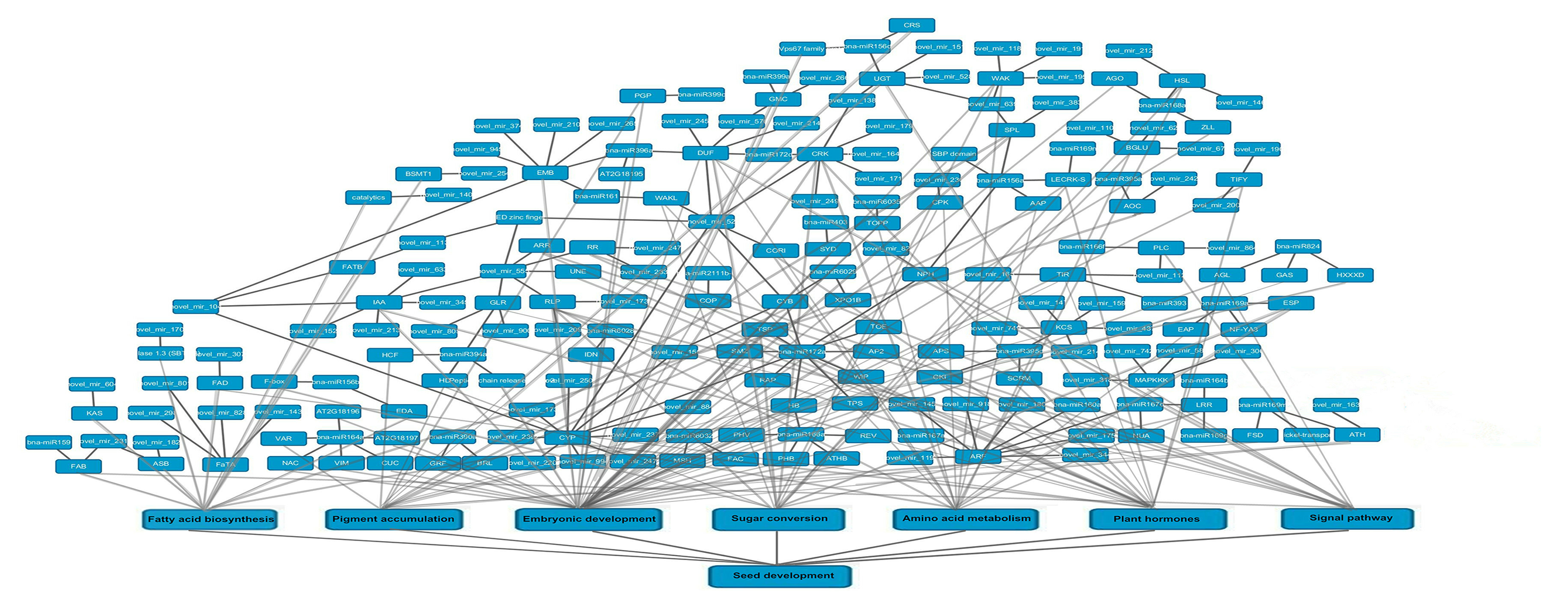

Supplement: Figure S2 — miRNA-mediated gene regulatory networks in developing seeds. [file Image2.TIF]

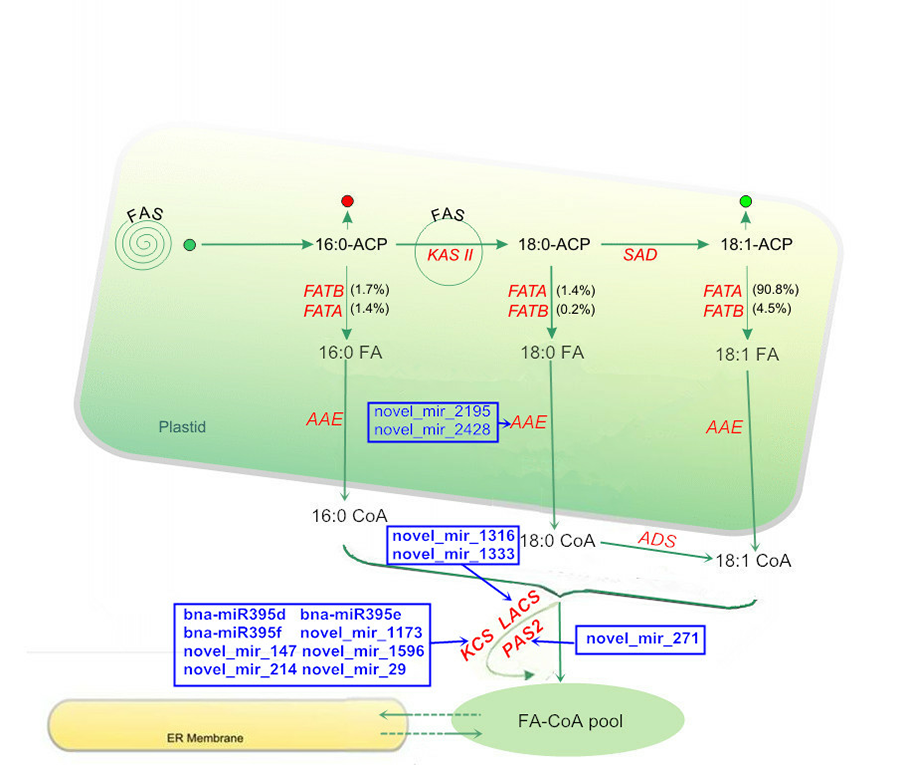

Supplement: Figure S3 — Analysis of pathways related to fatty acid desaturation and fatty acid elongation. The original drawings of fatty acid biosynthesis are from http://aralip.plantbiology.msu.edu/pathways/fatty_acid_synthesis. [file Image3.TIF]

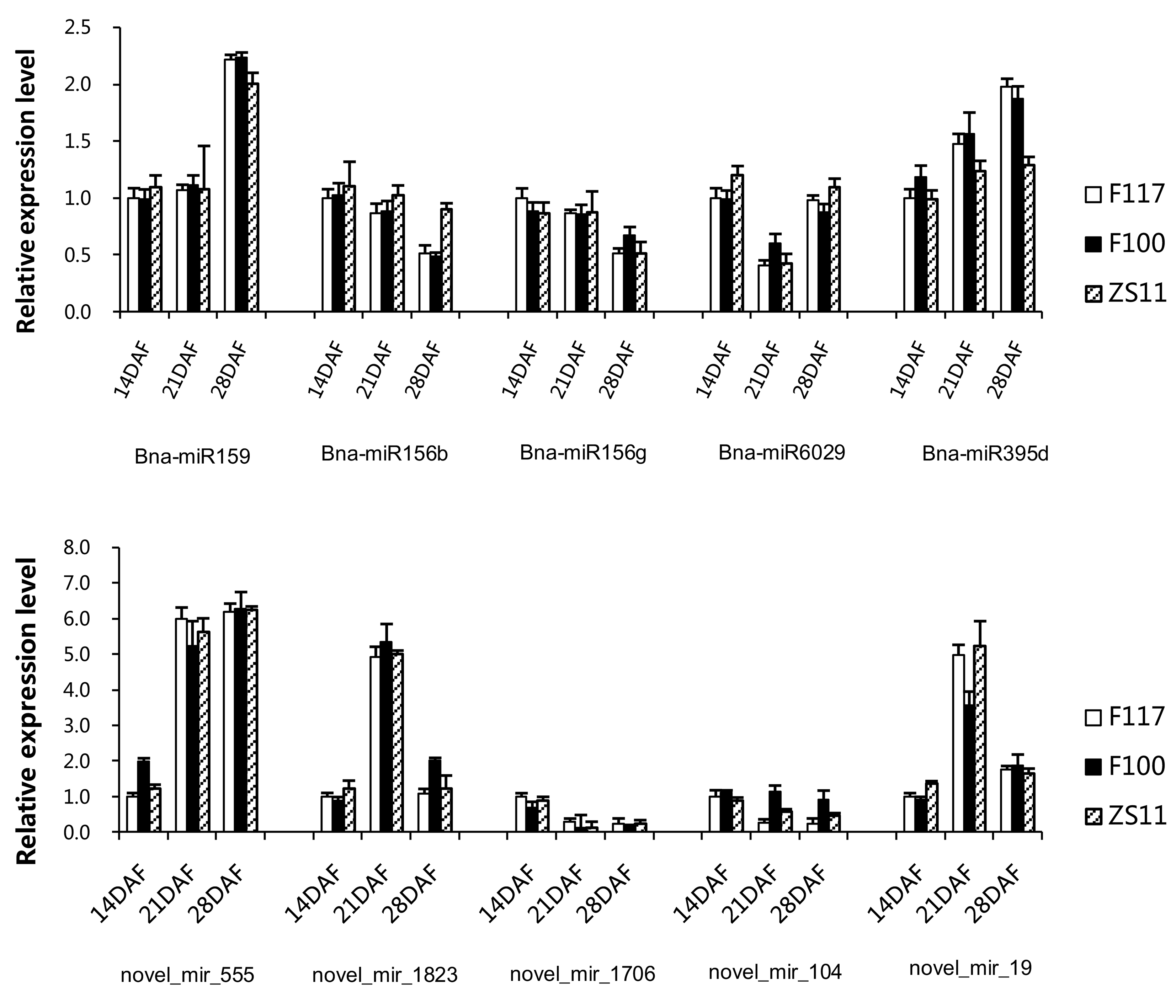

Supplement: Figure S4 — A biological replicates for qRT-PCR validation of selected miRNAs. F117, a B. napus double haploid line with stable oil content; F100, a B. napus double haploid line with unstable oil content; ZS11, zhongshuang11. [file Image4.TIF]
