# Supplementary material for: Identification of microRNAs Actively Involved in Fatty Acid Biosynthesis in Developing Brassica napus Seeds Using High-Throughput Sequencing
Source: Front Plant Sci. 2016 Oct 24;7:1570. doi: 10.3389/fpls.2016.01570 (PMC5075540; doi:10.3389/fpls.2016.01570)
Supplement: Table S1 — Raw data analysis for the three libraries. [file Table1.DOCX]

Table S1 Raw data analysis in three libraries

| category | 14DAF | 21DAF | 28DAF |
| --- | --- | --- | --- |
| total_reads | 12225750 | 11419839 | 11427691 |
| high_quality | 12182185(100%) | 11376189(100%) | 11384326(100%) |
| 3'adapter_null | 13400(0.11%) | 6425(0.06%) | 9107(0.08%) |
| insert_null | 2436(0.02%) | 1702(0.01%) | 3415(0.03%) |
| 5'adapter_contaminants | 28019(0.23%) | 28398(0.25%) | 21630(0.19%) |
| smaller_than_18nt | 17056(0.14%) | 3338(0.03%) | 10246(0.09%) |
| polyA | 1218(0.01%) | 1927(0.02%) | 4553(0.04%) |
| clean_reads | 12120056(99.49%) | 11334399(99.63%) | 11335373(99.57%) |
