# Supplementary material for: Identification of microRNAs Actively Involved in Fatty Acid Biosynthesis in Developing Brassica napus Seeds Using High-Throughput Sequencing
Source: Front Plant Sci. 2016 Oct 24;7:1570. doi: 10.3389/fpls.2016.01570 (PMC5075540; doi:10.3389/fpls.2016.01570)
Supplement: Table S6 — Primers used for mature miRNA qRT-PCR. [file Table6.DOCX]

Table S6 Primers used for mature miRNAs qRT-PCR

| miRNA name | Forward primer(5'-3') | R |
| --- | --- | --- |
| bna-miR156b | CTTGGCAGAAGAGAGTGAGCAC | Reverse primer |
| bna-miR156g | CTTGGCAGAAGAGAGTGAGCAC | Reverse primer |
| bna-miR159 | TGGATTGAAGGGAGCTCTAA | Reverse primer |
| bna-miR395d | CTGAAGTGTTTGGGGGGACTC | Reverse primer |
| bna-miR6029 | TGGGGTTGTGATTTCAGGCTT | Reverse primer |
| novel_mir_104 | GGAAGAAGAAGAAGAAGAAGCG | Reverse primer |
| novel_mir_555 | TCCAAGGAAGAAGAAGAAGATG | Reverse primer |
| novel_mir_173 | TCTTGTCGGAGTTTATGATC | Reverse primer |
| novel_mir_19 | GACCTGATTGCAATGATAACGG | Reverse primer |
| novel_mir_604 | GAGATGGCAATCATGGACTTG | Reverse primer |
| novel_mir_1407 | GATTAGTCGGTTGGGCTTCGGC | Reverse primer |
| novel_mir_1430 | GGATCGAATCCAGATCTCGGA | Reverse primer |
| novel_mir_1706 | GAGAGTTCGACGGCTAGGGT | Reverse primer |
| novel_mir_1823 | ACCATTTGGATTGGAGAAAGAGG | Reverse primer |
| novel_mir_1758 | GGAGGAGACGGAGGAGGAGGAG | Reverse primer |
| novel_mir_1081 | ATCAACCTGAACTGTTCGGTAT | Reverse primer |
| U6s | TTGGAACGATACAGAGAAGATTAGCA | Reverse primer |

Reverse primer was obtained from the miRcute miRNA qPCR detection kit (FP401, Tiangen, Beijing, China).
