# Supplementary material for: Identification of microRNAs Actively Involved in Fatty Acid Biosynthesis in Developing Brassica napus Seeds Using High-Throughput Sequencing
Source: Front Plant Sci. 2016 Oct 24;7:1570. doi: 10.3389/fpls.2016.01570 (PMC5075540; doi:10.3389/fpls.2016.01570)
Supplement: Table S7 — Primers used for predicted miRNA target qRT-PCR. [file Table7.DOCX]

Table S7 Primers used for predicted miRNAs targets qRT-PCR

| miRNA name | Target | Primer (from 5' to 3') | |
| --- | --- | --- | --- |
|  |  | F | R |
| bna-miR156b | BnaC01g44740D | TTGCCCAGACTCCTCCCT | ACGCCACTGCTCCTCCAT |
| bna-miR156g | BnaC05g33250D | CAGTATCCCTGTGGTATG | TTTGTGGTTGTTCTTGTT |
| bna-miR159 | BnaA06g13360D | TGCCTTGGAACTGGTCTG | AGTAATGTGGTTTGCCGC |
| bna-miR395d | BnaA06g04280D | AGCCACTCGTGCTTTCGT | ATAACCGCTTTGCCTCCC |
| bna-miR6029 | BnaA01g09630D | GCAAAAACAGTGAGCAAC | GTCATAGACACAAATACC |
| novel_mir_104 | BnaA03g37760D | GGATGGTTTTGTGATAGG | GGGGTAGATGTTGCGTGT |
| novel_mir_1407 | BnaA03g13780D | GCAGTATCTGGAGGTCTT | TATCTTCTGGATTGTGTC |
| novel_mir_1430 | BnaA03g37700D | CTGAATCTCATTTGGGTC | GCCGTCTTGTGTCTTGGT |
| novel_mir_1706 | BnaA06g06030D | AATCCACTGCCTCCCTTCCCTCT | CACCACACCTCCTATTCTCGTTC |
| novel_mir_173 | BnaA05g33500D | GTCTCTTTCCTTGGGTCC | GTGTGGGTTATTTGGTTT |
| novel_mir_1758 | BnaAnng14470D | TTGCCCAGACTCCTCCCT | ACGCCACTGCTCCTCCAT |
| novel_mir_1823 | BnaC05g14920D | TTACGACTGTTCCGCATTTCCCA | ACCACACCATCCTCCAACGCCTT |
| novel_mir_19 | BnaA01g21430D | AGGGTTGTAGACGAGGTT | GTAGGCGTTCACGGATGT |
| novel_mir_555 | BnaC01g40290D | TACTCCTCACATCTTCCA | GGCTATCTTCTCCACAAC |
| novel_mir_604 | BnaC01g32050D | AATAAAAAAGGTAAAATG | GTGGAGAAGCACAGAGAG |
| novel_mir_1081 | BnaC02g41820D | AGAACTGCCGTCGTTACTG | GATCCTGCTTAGGCTTGGA |
|  | Actin | TGGGTTTGCTGGTGACGAT | TGCCTAGGACGACCAACAATACT |
